# Anti-Phosphatidylserine/Prothrombin Antibodies Are Associated with Adverse Pregnancy Outcomes

**DOI:** 10.1155/2015/975704

**Published:** 2015-05-20

**Authors:** Polona Žigon, Katja Perdan Pirkmajer, Matija Tomšič, Tanja Kveder, Borut Božič, Snežna Sodin Šemrl, Saša Čučnik, Aleš Ambrožič

**Affiliations:** ^1^Department of Rheumatology, University Medical Centre Ljubljana, Vodnikova 62, SI-1000 Ljubljana, Slovenia; ^2^Faculty of Pharmacy, University of Ljubljana, Aškerčeva cesta 7, SI-1000 Ljubljana, Slovenia; ^3^Faculty of Mathematics, Natural Science and Information Technology, University of Primorska, SI-6000 Koper, Slovenia

## Abstract

*Objective*. To determine the prevalence and clinical association of anti-phosphatidylserine/prothrombin antibodies (aPS/PT) in patients with a history of pregnancy complications relevant to antiphospholipid syndrome (APS). *Materials and Methods*. Two hundred and eleven patients with a history of (a) three or more consecutive miscarriages before 10th week of gestation (WG) (*n* = 64), (b) death of a morphologically normal fetus beyond 10th WG (*n* = 72), (c) premature birth of a morphologically normal neonate before 34th WG due to eclampsia, preeclamsia and placental insufficiency (*n* = 33), and (d) less than three unexplained consecutive miscarriages before 10th WG (*n* = 42). Subjects sera were analyzed for lupus anticoagulant (LA), anti-cardiolipin (aCL), anti-*β*_2_-glycoprotein I (anti-*β*_2_GPI), and aPS/PT antibodies. *Results*. 41/169 (24.3%) of patients were positive for at least one measured aPL. The highest prevalence was found for aPS/PT and aCL (13.0% and 12.4%, resp.) followed by LA (7.7%) and anti-*β*_2_GPI (7.1%). 11/169 with APS-related obstetric manifestations had only aPS/PT. 17.8% of patients were positive for LA or aCL and/or anti-*β*_2_GPI; however when adding the aPS/PT results, an additional 7% of patients could be evaluated for APS. *Conclusion*. aPS/PT are associated with recurrent early or late abortions and with premature delivery irrespective of other aPL.

## 1. Introduction

Patients with elevated levels of antiphospholipid antibodies (aPL) often experience pregnancy complications comprising recurrent spontaneous abortions, intrauterine growth retardation, and preeclampsia, suggesting that these antibodies may influence embryonic implantation and induce thrombosis of the uteroplacental vasculature. The international classification criteria for antiphospholipid syndrome (APS) connect the occurrence of obstetric complications and/or thrombosis together with persistence of aPL in APS [1]. Laboratory criteria for APS include lupus anticoagulants (LA), anticardiolipin antibodies (aCL), and antibodies against *β*_2_-glycoprotein I (anti-*β*_2_GPI). Several other “noncriteria” aPL are believed to be associated with APS; however the lack of evidence confirming their diagnostic applicability so far prevents their inclusion into classification criteria. In recent years, many studies demonstrated the association of antiprothrombin antibodies with the pathogenesis of APS [2–4] and some of them proposed their beneficial role for APS diagnosis [5–7]. These antibodies can be detected by an ELISA targeting prothrombin alone (aPT-A) or targeting phosphatidylserine/prothrombin complex (aPS/PT) [8]; however the latter are more frequently found in patients with APS [4, 9, 10]. Our group reported on an* in-house* aPS/PT ELISA as the optimal method for the determination of clinically relevant antiprothrombin antibodies exhibiting the highest proportion of LA in our population of patients [11]. Clinical relevance of antiprothrombin antibodies was mainly described for patients with APS and thrombosis but very few studies reported their association with adverse pregnancy outcomes. A comprehensive review of “antiprothrombin antibodies” and “pregnancy/obstetric/miscarriages/fetal loss” revealed 12 studies, comprising 1031 patients and 988 controls (Table 1). Half of these studies failed to find any significant association between antiprothrombin antibodies and pregnancy morbidities [12–16]. On the contrary, Akimoto et al. [17] presented strong and specific association between various types of antiprothrombin antibodies with severe preeclampsia and spontaneous abortion. Only the study from Bertolaccini et al. [18] differentiated among different obstetric complications and showed a significant association of both aPS/PT and aPT-A with unexplained death of a morphologically normal fetus beyond 10th week of gestation. The clinical significance of antiprothrombin antibodies in patients with adverse pregnancy outcome was later confirmed also by Marozio et al. [3], who investigated aPT-A, and Vlagea et al. [19] who investigated aPS/PT. Furthermore, our group has recently reported that aPS/PT is the strongest independent risk factor for obstetric complications compared to LA, aCL, and anti-*β*_2_GPI [20]. However, no reports to date were found describing association of antiprothrombin antibodies with unexplained consecutive miscarriages in the first trimester of pregnancy.

Therefore, the present study aims to investigate the association of aPS/PT with a history of specific idiopathic pregnancy complications in a larger group of patients and to determine whether the presence of aPS/PT is associated with an increased risk of obstetric manifestations relevant for APS.

## 2. Materials and Methods

### 2.1. Subjects

This study included 402 sera samples which were prospectively collected from 211 consecutive female patients (median age 33 years, IQR: 7 years) referred between 2005 and 2013 to our clinic due to possible obstetric APS (Table 2). Among them 169 had obstetric manifestations included in the APS classification criteria. Specifically, 64 patients had three or more unexplained consecutive miscarriages before 10th week of gestation (WG), 72 patients experienced unexplained death of a morphologically normal fetus past 10th WG, and 33 patients gave premature birth to a morphologically normal neonate before 34th WG due to eclampsia, preeclamsia, or placental insufficiency. Among 33 preterm deliveries, 10 cases experienced fetal death as a result of either placenta abruptions or growth retardations with any morphological abnormalities being excluded. There were 16 cases with induced deliveries because of preeclampsia or eclampsia and 7 spontaneous preterm deliveries. The remaining 42 patients experienced less than three miscarriages before 10th WG and did not meet the APS classification criteria.

All patients and their partners were investigated for genetic abnormalities. The majority of patients had received the results of genetic analysis at the time of their visit to our clinic and the patients with confirmed abnormalities were excluded from the study.

As a control group we included 87 healthy female blood donors (median age 42 years, IQR: 18 years) without a history of underlying autoimmune disease, bleeding disorders, thrombosis, and/or pregnancy morbidity.

All patients had their sera collected during their clinical examination in the Department of Rheumatology (University Medical Centre, Ljubljana). The samples were aliquoted, stored at −20°C, and subsequently analyzed. This study was conducted as part of the National Research Program “Systemic Autoimmune Diseases” (#P3-0314). Participants signed an informed consent and the study was approved by the National Medical Ethics Committee, Ljubljana, Slovenia.

### 2.2. LA Determination

Plasma samples were analyzed using coagulation analyzer BCS Siemens according to the guidelines valid at the time the study started [22]. Simplified Dilute Russell's Viper Venom Test (dRVVT) was performed using LA 1 screening reagent and LA 2 confirmatory reagent (Siemens) following manufacturer's instructions [23]. A dRVVT ratio (LA1 screen/LA2 confirmation) above 1.2 was considered positive for LA activity. Activity of LA was quantified as follows: low positive (LA1/LA2 = 1.2–1.5), medium (LA1/LA2 = 1.5–2.0), and high positive (LA1/LA2 > 2.0).

### 2.3. An In-House aPS/PT ELISA

This was performed following previously described protocol and validated method [11]. Specifically, the assays average inter- and intra-assay coefficients of variations were <3.3% and <8.2%, respectively. The diagnostic specificity for APS was 92.5% and the diagnostic sensitivity was 59.0%. The diagnostically relevant cut-off of aPS/PT was set on the 99th percentile of 222 blood donors. Briefly, phosphatidylserine was coated on polystyrene microtitre plates (medium binding, Costar, Cambridge, MA, USA) and incubated overnight at 4°C. After blocking with Tris-buffered saline (TBS) containing 1% bovine serum albumin (BSA) and 5 mM CaCl_2_ (1% BSA-TBS-Ca^++^) plates were washed in TBS containing 0.05% Tween-20. Human prothrombin (10 mg/L) (Enzyme Research Laboratories, UK) and patients' sera diluted 1 : 100 in 1% BSA-TBS-Ca^++^ were applied to wells immediately one after the other and incubated for 1 hour at room temperature (RT). Afterwards, plates were washed and incubated with alkaline phosphatase-conjugated goat anti-human IgG or IgM (ACSC, Westbury, USA) for 30 minutes at RT. After the last wash para-nitrophenylphosphate (Sigma Chemical Company, USA) in diethanolamine buffer (pH 9.8) was applied as substrate and OD_405_ was kinetically measured by microtitre plate reader (Tecan, Grödig, Austria).

### 2.4. IgG and IgM aCL

These were determined in sera by an* in-house* solid phase aCL ELISA [24]. Briefly, polystyrene microtitre plates (medium binding, Costar, Cambridge, MA, USA) were coated with cardiolipin (Sigma, St. Louis, USA) and blocked with 10% fetal bovine serum (FBS) (Sigma, St. Louis, USA) in phosphate-buffered saline (PBS). After washing with PBS, diluted samples in 10% FBS-PBS were applied and incubated for 2.5 hours at RT. The detection system was the same as in aPS/PT ELISA.

### 2.5. IgG and IgM Anti-**β**_2_GPI

These were measured by an* in-house* ELISA [25]. Briefly, high binding polystyrene microtitre plates coated with 50 uL/well of *β*_2_GPI (10 mg/L) in PBS were incubated for two hours at RT. The plates were then washed with PBS containing 0.05% Tween-20 (PBS-Tween) and incubated with samples diluted in PBS-Tween for 30 minutes at RT. The detection system was the same as in aPS/PT ELISA.

All aPL tests were performed in the same sera samples. Patients and controls were tested at the same time. None of the patients or controls were pregnant at the time of aPL determination.

### 2.6. Statistical Analysis

Statistical analysis was performed using the SPSS 15.0 program. The receiver operating characteristic (ROC) analysis and the area under the curve (AUC) were used to assess the diagnostic performance of the measured marker(s). The results of multivariate logistic models were approximated by odds ratio with its 95% confidence interval (OR [95%]). A 2-sided *P* value < 0.05 was considered statistically significant.

## 3. Results

Every patient positive for any of the tested aPL was tested again at least 12 weeks after their first visit and only permanently elevated levels of aPL were considered as a positive result. Prevalence of all aPL tested is shown in Table 3. Overall, 169 patients experienced pregnancy morbidity defined by APS criteria and 41 (24%) of them showed permanent positivity for at least one of the measured aPL. The highest prevalence was found for aCL and aPS/PT (13%) while the prevalence for LA and anti-*β*_2_GPI was lower (7%) (Table 3). Eleven patients (6.5%) were aPS/PT positive while being negative for all other tested aPL. Six of them had recurrent abortions before 10th WG, two experienced an unexplained death of a morphologically normal fetus past 10th WG, one delivered prematurely, and two experienced both recurrent abortions and premature birth. Considering Sydney revised laboratory criteria of APS, 17.8% (30/169) of patients were positive for LA or aCL and/or anti-*β*_2_GPI. Among them, 22 were treated with low-molecular-weight heparin (LMWH) and low dose aspirin (LDA) which resulted in a successful pregnancy. Six women had no subsequent pregnancies; three of them experienced successful pregnancy prior to APS pregnancy complications. Two women had unsuccessful subsequent single pregnancies despite the anticoagulant treatment. When adjoined, the aPS/PT results revealed an additional 6.5% (11/169) of patients with adverse pregnancy outcome that was positive for aPL.

The frequency of aPL among 42 patients who experienced less than three miscarriages before 10th WG (and did not meet APS classification criteria) was very low and did not show differences from healthy women.

Statistical analyses could not find any association of higher levels of aPL being more strongly associated to adverse pregnancy outcome.

When analyzing each of the three categories of pregnancy morbidity included in the APS classification criteria only aPS/PT antibodies were statistically significantly associated with either of three types of adverse pregnancy outcome (*P* < 0.03, age adjusted) (Table 4). In fact, these are the only aPL with the significantly higher frequency in patients experiencing recurrent abortion before 10th WG compared to healthy women. On the contrary, anti-*β*_2_GPI antibodies showed no association with any kind of pregnancy morbidity. aCL and LA were associated with obstetric complications appearing late in pregnancy; however they did not show any association with early pregnancy morbidity.

Age-adjusted analyses were performed in order to estimate the relative risk of positive outcome in different aPL tests (LA, aCL, anti-*β*_2_GPI, and aPS/PT) to obstetric complications characteristic for APS presented as OR with 95% confidence interval. As shown in Table 5, only aCL and aPS/PT antibodies presented an elevated risk for obstetric complications (OR 7.4 [95% CI 1.6–34.5] and OR 7.4 [95% CI 1.5–35.2], resp.).

In our group of 169 patients, 12 (6%) had a history of thrombosis and the prevalence of all tested aPL was higher among them as compared to healthy controls (*P* < 0.01). Excluding these 12 patients from logistic regression analyses showed that frequencies of aCL and aPS/PT antibodies were still significantly higher as compared to healthy controls (*P* = 0.04 and *P* = 0.015, resp.).

## 4. Discussion

While several studies evaluated aPL positivity in APS patients with a history of thrombosis only a few studies established an association of different aPL with individual obstetric abnormalities distinctive for APS. The question arises whether the same profile of aPL occurs in APS patients with a history of thrombosis compared to obstetric APS. There is general consensus to screen for LA, aCL, and anti-*β*_2_GPI, but the role of other autoantibodies remains controversial. Therefore, the necessity to perform more cohort studies in order to determine the incidence of noncriteria aPL in pregnancy loss was suggested [26]. Since then, our group focused on evaluating the prevalence of aPS/PT antibodies among female patients experiencing different obstetric complications during their pregnancies.

We found an overall prevalence of aPS/PT of 13.0%, aCL of 12.4%, LA, and anti-*β*_2_GPI less than 8.0% in our group of patients with obstetric complications characteristic for APS. Both aPS/PT and aCL were significantly more prevalent in our cohort of patients compared to healthy blood donors. However, aCL correlated only with late pregnancy morbidity and prematurity while aPS/PT were the only antibodies associated with early recurrent pregnancy loss, as well as with late pregnancy morbidity and prematurity. Our findings are in line with Clark et al. [27] who suggested that aCL-associated early recurrent pregnancy loss be withdrawn from the classification criteria due to inconsistent prevalence of aCL in this population and an increasing body of evidence points to the fact that this clinical manifestation of APS is distinct from late loss or early delivery with placental infarction. However, according to our results, determining aPS/PT in association with early recurrent pregnancy loss may be beneficial. We found 7% (11/169) of patients to be aPS/PT positive and negative for all other tested aPL and 63% (7/11) of them experienced early recurrent pregnancy loss. Only one published study also confirmed association of antiprothrombin antibodies to early pregnancy loss; however it differed from the current study in that they measured aPT-A and included patients whose pregnancies ended spontaneously within 20th WG [21]. Considering the Sydney revised laboratory criteria of APS, only 17.8% (30/169) of patients in our study were positive for LA or aCL and/or anti-*β*_2_GPI, but when aPS/PT was evaluated as an additional parameter, 24.8% (41/169) of patients were aPL positive.

We have also determined the frequency of aPL among 42 patients who experienced less than 3 miscarriages before 10th WG and did not meet APS classification criteria. In line with our expectations the prevalence of all aPL in this group was very low (<7%) and was not different from frequencies found in healthy women.

The review of the literature revealed 12 studies dated from 1997 to 2013. More than half of the articles were published before 2006, when new revised classification criteria were accepted. Five studies tested only aPT-A antibodies, which are (according to newer data) less significant for APS [3, 12, 14, 17, 21]. Among five larger studies, recruiting more than 100 patients, three showed significant association of aPS/PT or aPT-A with obstetric complications [3, 18, 21]; one did not find any significant association; however they only measured aPT-A antibodies [14], and one was performed before 2006 and recruited patients with more than two recurrent miscarriages, instead of three [15]. Conflicting data in the literature and the lack of studies investigating the role of antiprothrombin antibodies in obstetric APS led us to study the occurrence of these antibodies in different early and late adverse pregnancy outcomes. The overall prevalence of aPS/PT antibodies in our study was 13.0%. This number is higher than the ones reported by Andreoli et al. [28] who reviewed 120 studies investigating LA, aCL, and anti-*β*_2_GPI and provided an overall aPL frequency estimated as 6% for pregnancy morbidity. Rare studies investigated clinical significance of antiprothrombin antibodies in relation to adverse obstetric outcome. Akimoto et al. [17] found the mean titer of antiprothrombin-1 antibodies in patients with spontaneous abortions to be greater than in normal pregnant women. However, subsequent studies failed to show any association of antiprothrombin antibodies (aPT or aPS/PT) with early recurrent pregnancy loss [14–16]. Bertolaccini et al. [18] found correlation between aPS/PT and fetal death beyond 10th WG but not to early pregnancy loss or to prematurity. Marozio et al. [3] reported a positive correlation between aPT-A and adverse late pregnancy outcome. Two recent studies [19, 20] found correlation between aPS/PT and obstetric abnormalities; however neither specified the types of pregnancy complications.

Our study did not confirm the association of either anti-*β*_2_GPI or LA with any of the adverse pregnancy outcomes. This is in line with conclusions of the systematic review [29], implying that there is currently insufficient data to establish any significant link between anti-*β*_2_GPI and pregnancy morbidity. Also, in the study by Clark et al. [30], which included 2257 women attending a high-risk pregnancy clinic, less than 1% of patients with early recurrent miscarriage tested positive for LA, while on the other hand patients positive for LA had a significantly more frequent history of thrombosis. Possibly, LA and anti-*β*_2_GPI are far more associated with thrombotic APS than obstetric APS, while aCL and aPS/PT appear to be associated with both types of APS.

The diagnostic accuracy of individual aPL for pregnancy losses defined by APS classification criteria, determined by AUC, was low, ranging from 0.499 for anti-*β*_2_GPI to 0.549 for aCL or aPS/PT (Table 5). In our previous study [20], AUC for different aPL and APS (either thrombotic or obstetric) varied from 0.88 for IgG aCL to 0.55 for IgM anti-*β*_2_GPI, while Otomo et al. determined AUC (for the revised Sydney criteria) to be 0.688 [31]. Certain patients with clinical symptoms significant for APS, fulfilling clinical criteria, are negative for all tested aPL and within this group aPS/PT despite lower AUC could improve the diagnoses of APS increasing sensitivity of the total aPL.

So far, the mechanism by which antiprothrombin antibodies might be involved in morbidity during pregnancy has not been clarified. However, it is very likely that there are more possible pathways involved. It has been suggested that they may lead to pregnancy loss by the promotion of microvascular placental thrombosis, which was supported by histological findings [32]. Placental trophoblast is the main organ in which phosphatidylserine is highly exposed on the outer leaflet of the membrane. During embryonic and placental differentiation a disruption of the lipid asymmetry occurs, leading to exposure of phosphatidylserine on the outer surface [33]. Antiprothrombin antibodies might crosslink prothrombin on the cell surface where they could complicate pregnancy by complement activation or by interfering with signaling which seems to be an essential factor for disease manifestation from the results of the* in vivo* experiments [34]. An increased number of apoptotic events of giant cells in the phosphatidylserine-exposed ectoplacenta were observed, which may lead to insufficient development of the placenta resulting in embryo small for date or fetal loss [35]. On the other hand, it has been observed that, in mice, prothrombin plays an important role in the development of the embryo and that prothrombin deficiency results in embryonic and neonatal lethality [36]. There is no doubt that clarification of the pathways in which antiprothrombin antibodies are involved in the pathogenesis of APS needs to be further investigated, but nevertheless it has been shown that patients treated for APS have good pregnancy outcomes [37]. A review by Marchetti et al. [38] concluded that screening for high-risk APS patients is necessary to improve their pregnancy outcome, and we showed that aPL profile screening including aPS/PT, in addition to LA, aCL, and anti-*β*_2_GPI, could enable better evaluation of high-risk APS patients and possibly predict further pregnancy losses. Similarly, Ulcova-Gallova et al. [39] suggested that determination of aPL only against cardiolipin in patients with reproductive failure is not sufficient for obstetric-gynecology diagnosis; therefore the investigation of aPS/PT in this group of patients could be warranted.

## 5. Conclusion

aPS/PT are associated with adverse pregnancy outcome irrespective of other antiphospholipid antibodies. Therefore, aPS/PT measurement might improve the evaluation of patients with early recurrent pregnancy loss, undiscovered by other aPL tests. Further studies including a larger number of patients with pregnancy complications and/or reproductive failure and apparently healthy donors are needed to determine the independent effects of various antiphospholipid antibodies, as well as aPS/PT, and confirm their potential use in clinical practice.

## Figures and Tables

**Table 1:** Review of antiprothrombin antibodies and pregnancy complications.

Author, year (ref.)	Study design	Number of controls (feature)	Number of patients (event)	ELISA	Isotype	Sensit. (%)	OR	*P*
Forastiero et al., 1997 [12]	R	89 (no obstetric complications)	44 (obstetric complications)	aPT-A	IgG	20	1.4	ns
					IgM	12	1.8	ns

Akimoto et al., 2001 [17]	P	12 (healthy nonpregnant)	19 (abortion <13th WG)	aPT-A	**IgG**	**58**		**<0.01**
		36 (normal pregnancy)	28 (severe preeclampsia)	aPT-A	**IgG**	**36**		**<0.01**

Tsutsumi et al., 2001 [16]	R		81 (≥2 recurrent miscarriages)	aPS/PT	IgG	0		
					IgM	0		

Sugiura-Ogasawara et al., 2004 [15]	R		100 (≥2 recurrent miscarriages)	aPS/PT	IgG	1		
					IgM	0		

Bertolaccini et al., 2005 [18]	R(M)	71 (healthy)	40 (recurrent abortions <10th WG)	aPS/PT	IgG	12	1.2	ns
					IgM	9	1.4	ns
				aPT-A	IgG	11	0.9	ns
					IgM	0	0.2	ns
			37 (fetal death >10th WG)	aPS/PT	**IgG**	**17**	**3.3**	**0.005**
					**IgM**	**13**	**3.7**	**0.006**
				aPT-A	**IgG**	**19**	**4.1**	**0.0007**
					IgM	1	0.5	ns
			29 (prematurity <34th WG preeclampsia/eclampsia/placental insufficiency)	aPS/PT	IgG	9	1.2	ns
					IgM	5	0.9	ns
				aPT-A	IgG	10	1.4	ns
					IgM	0	0.3	ns

Nojima et al., 2006 [13]	R(M)	74 (healthy)	14 (fetal loss)	aPS/PT		21	1.49	ns
				aPT-A		14	0.25	ns

Sabatini et al., 2007 [21]	RCC	200 (pregnant, gestational/age matched)	100 (pregnant, past ≥3 recurrent abortions <20th WG)	aPT-A	**IgG**	**37**		**<0.001**
					**IgM**	**18**		**<0.001**
					IgA	4		ns

Marozio et al., 2011 [3]	RCC	163 (uneventful pregnancy)	163 (adverse late pregnancy outcome)	aPT-A	**IgG**	**26**	**9.1**	**<0.001**
					IgM	2	na	ns

Sater et al., 2012 [14]	RCC	288	277 (≥3 miscarriages <12th WG)	aPT-A	IgM	4	14.27	ns

Vlagea et al., 2013 [19]	R	/	71 (obstetric abnormalities)	aPS/PT	**IgG**	**25**	**2.37**	**0.04**
					IgM	27	1.32	ns

Žigon et al., 2013 [20]	R(M)	55 (no obstetric complications)	28 (APS obstetric abnormalities)	aPS/PT	**IgG**	**64**	**9.3**	**<0.001**
					**IgM**	**54**	**4.0**	**<0.005**

Medline query with keywords “antiprothrombin antibodies” and “pregnancy/obstetric/miscarriages/fetal loss” in various combinations yielded the documented reports. R: retrospective study, R(M): retrospective study with multivariate analyses, P: prospective study, RCC: retrospective case control study, ns: not significant, WG: week of gestation, OR: odds ratio, and Sensit.: diagnostic sensitivity.

**Table 2:** Patients' clinical features.

Women with obstetric complications *n* = 211 (%)	
Thrombosis	12 (6)
Arterial thrombosis	8 (4)
Venous thrombosis	4 (2)
Pregnancy loss defined by APS criteria [1]	169 (80)
≥3 consecutive miscarriages <10th WG	64 (30)
Fetal death >10th WG	72 (34)
Premature birth <34th WG	33 (16)
Pregnancy loss not defined by APS criteria	42 (20)
<3 miscarriages <10th WG	42 (20)

**Table 3:** Prevalence of aPL in patients with obstetric complications and healthy controls.

	Healthy controls *n* = 87 (%)	Non-APS obstetric manifestation *n* = 42 (%)	Pregnancy loss defined by APS criteria [1] *n* = 169 (%)
LA	0	2 (5.3)^*^	13 (8.7)^**^
aCL	2 (2.3)	2 (4.8)	21 (12.4)
IgG	1 (1.2)	2 (4.8)	17 (10.1)
IgM	1 (1.2)	0	6 (3.6)
anti-*β*_2_GPI	6 (6.9)	3 (7.1)	12 (7.1)
IgG	6 (6.9)	3 (7.1)	10 (5.9)
IgM	0	0	4 (2.4)
aPS/PT	2 (2.3)	0	22 (13.0)
IgG	1 (1.2)	0	16 (9.5)
IgM	1 (1.2)	0	12 (7.1)

^*^(*n* = 38), ^**^(*n* = 149).

aCL: anticardiolipin antibody, anti-*β*_2_GPI: antibodies against *β*_2_-glycoprotein I, aPS/PT: anti-phosphatidylserine/prothrombin antibodies, and LA: lupus anticoagulant.

**Table 4:** Diagnostic accuracy of aPL for different adverse pregnancy outcomes.

	≥3 consecutive miscarriages <10th WG			Fetal death >10th WG			Premature birth <34th WG		
	AUC	OR [95% CI]	*P*	AUC	OR [95% CI]	*P*	AUC	OR [95% CI]	*P*
LA	0.508	1.0 [1.0-1.1]	ns	0.563	1.1 [1.1-1.2]	0.001	/	/	/
aCL	0.516	2.8 [0.5–16.0]	ns	0.572	8.3 [1.8–37.6]	0.002	0.562	9.4 [1.8–49.6]	0.002
IgG	0.512	4.2 [0.4–41.6]	ns	0.558	12.6 [1.6–100]	0.003	0.568	19.1 [2.2–165]	<0.001
IgM	0.503	1.4 [0.1–22.2]	ns	0.52	4.2 [0.5–38.3]	ns	0.494	0.7 [0.7-0.8]	ns
anti-*β*_2_GPI	0.501	0.9 [0.2–3.3]	ns	0.497	0.8 [0.3–2.8]	ns	0.465	0.8 [0.2–4.5]	ns
IgG	0.492	0.7 [0.2–2.8]	ns	0.491	1.1 [0.4–3.6]	ns	0.465	0.9 [0.2–4.6]	ns
IgM	0.509	0.4 [0.4-0.5]	ns	0.513	0.5 [0.4–0.6]	ns	/	/	/
aPS/PT	0.534	5.3 [1.1–26.2]	0.026	0.559	6.8 [1.5–31.4]	0.005	0.544	7.5 [1.4–40.8]	0.008
IgG	0.531	9.0 [1.1–76.3]	0.017	0.533	7.5 [0.9–62.6]	0.030	0.550	15.2 [1.7–135]	0.002
IgM	0.512	4.3 [0.4–42.3]	ns	0.533	7.6 [0.9–63.3]	0.029	0.513	8.6 [0.9–85.9]	0.030

AUC: area under the curve, aCL: anticardiolipin antibody, anti-*β*_2_GPI: antibodies against *β*_2_-glycoprotein I, aPS/PT: anti-phosphatidylserine/prothrombin antibodies, CI: confidence interval, LA: lupus anticoagulant, ns: not significant, OR: odds ratio, and WG: week of gestation.

**Table 5:** Diagnostic accuracy of aPL for pregnancy loss as defined by APS classification criteria.

	Pregnancy loss defined by APS criteria [1]		
	AUC	OR [95 %CI]	*P*
LA	0.541	0.6 [0.6-0.7]	ns
aCL	0.549	7.4 [1.6–34.5]	0.010
IgG	0.541	12.1 [1.5–97.5]	0.019
IgM	0.514	2.8 [0.3–26.9]	ns
anti-*β*_2_GPI	0.499	1.5 [0.5–4.6]	ns
IgG	0.492	1.2 [0.4–3.9]	ns
IgM	0.514	0.7 [0.6-0.7]	ns
aPS/PT	0.549	7.4 [1.5–35.2]	0.012
IgG	0.535	11.0 [1.3–91.5]	0.012
IgM	0.525	9.0 [1.0–78.9]	0.047

AUC: area under the curve, aCL: anticardiolipin antibody, anti-*β*_2_GPI: antibodies against *β*_2_-glycoprotein I, aPS/PT: anti-phosphatidylserine/prothrombin antibodies, CI: confidence interval, LA: lupus anticoagulant, ns: not significant, and OR: odds ratio.

## Abbreviations

anti-*β*_2_GPI: Antibodies against *β*_2_-glycoprotein I
aCL: Anticardiolipin antibodies
APS: Antiphospholipid syndrome
aPS/PT: Phosphatidylserine dependent antiprothrombin antibodies
aPT: Antibodies against prothrombin alone
AUC: Area under the curve
CI: Confidence interval
LA: Lupus anticoagulant
OR: Odds ratio
PS: Phosphatidylserine
ROC: Receiver operating characteristic.
